# A novel peptide specifically binding to VEGF receptor suppresses angiogenesis *in vitro* and *in vivo*

**DOI:** 10.1038/sigtrans.2017.10

**Published:** 2017-05-12

**Authors:** Yuan Zhang, Bifang He, Kun Liu, Lin Ning, Delun Luo, Kai Xu, Wenli Zhu, Zhigang Wu, Jian Huang, Xun Xu

**Affiliations:** 1 Department of Ophthalmology, Shanghai General Hospital, Shanghai Jiaotong University School of Medicine, Shanghai, China; 2 Key Laboratory for NeuroInformation of Ministry of Education, School of Life Science and Technology, University of Electronic Science and Technology of China, Chengdu, China; 3 Chengdu Nuoen Biotechnologies, LTD, Chengdu, China; 4 Center for Informational Biology, University of Electronic Science and Technology of China, Chengdu, China

## Abstract

Vascular endothelial growth factor (VEGF), one of the most important angiogenic factors, plays an essential role in both physiological and pathological angiogenesis through binding to VEGF receptors (VEGFRs). Here we report a novel peptide designated HRHTKQRHTALH (peptide HRH), which was isolated from the Ph.D. -12 phage display library using VEGFR-Fc fusion protein as the bait. This peptide was found to dose-dependently inhibit the proliferation of human umbilical vein endothelial cells stimulated by VEGF. The anti-angiogenesis effect of the HRH peptide was further confirmed *in vivo* using the chick chorioallantoic membrane assay, which was also dose-dependent. Besides, peptide HRH was proved to inhibit corneal neovascularization in an alkali-burnt rat corneal model and a suture-induced rat corneal model. Taken together, these findings suggest that the HRH peptide can inhibit angiogenesis both *in vitro* and *in vivo*. Consequently, the HRHTKQRHTALH peptide might be a promising lead peptide for the development of potential angiogenic inhibitors.

## Introduction

The deregulated and persistent angiogenesis is a critical hallmark of neovascular or exudative age-related macular degeneration (AMD).^1^ Overwhelming evidence has reported that abnormal angiogenesis is involved in many other pathological disorders including cancer, which are termed as angiogenesis-dependent diseases.^2^ Anti-angiogenesis treatment has become the standard-of-care therapy for angiogenesis-induced complications such as neovascular AMD and retinopathy of prematurity.^3,4^

Vascular endothelial growth factor (VEGF) is a pivotal angiogenic factor, which signals through its receptors on the cell surface to manipulate multiple facets of angiogenesis.^5^ The upregulated expression of VEGF is often present in patients with angiogenesis-related pathological disorders. Hence, the inhibition of VEGF has been already applied widely in the treatment of intraocular neovascular disorders. In fact, a large number of anti-VEGF agents have been developed.^6–9^ Many clinical trials have shown that the injection of anti-VEGF drugs has significant curative effects on ocular diseases driven by abundant angiogenesis.^10–15^ However, these existing therapeutic drugs are not effective to all patients, and many show decreased responsiveness and drug resistance in the course of therapy.^16^ Another problem is the consecutive intravitreal injections of drugs are costly and risky. Therefore, more new drugs that respond to more patients and, particularly, agents that would produce curative efficacy when administered by safer and simpler ways are needed urgently in treating AMD and other angiogenesis-dependent diseases.

Currently, most angiogenic inhibitors in clinical use are proteins, and a few are small molecules.^6^ Small-molecule therapeutics may sustain reduced target selectivity because of their fairly small size, whereas protein drugs are inclined to have high target specificity owning to more interactions between them, however this comes at the expense of poor membrane permeability, metabolic instability and low bioavailability.^17^ Fortunately, peptide agents have the advantages of both small molecules (low cost, oral bioavailability, membrane permeability, metabolic stability and conformational restriction) and proteins (target specificity and high potency). Also, to optimize efficacy and enhance metabolic stability, chemical modifications can be integrated into peptide synthesis, which can improve druggability.^18^ Therefore, compared with therapeutic proteins, synthetic peptides have several advantages as drug candidates. Phage display technology was first described by George P Smith in 1985,^19^ which has been extensively used for the identification of peptide ligands with high specificity for a given target.^20–24^ This approach has a few advantages over conventional random screening methods in drug discovery: less time-consuming, cost-effective and easy to operate. Giordano *et al.*^25^ adopted a phage-displayed peptide library-screening approach and identified the peptide CPQPRPLC specifically binding to VEGF receptor-1 (VEGFR-1) and neuropilin-1. Very recently, the derived peptidomimetic [_D_(CLPRC)], also known as Vasotide, has been reported to reduce retinal angiogenesis through either eye drops or intraperitoneal injection into three animal models.^26^

To obtain more novel anti-angiogenesis peptide ligands, in the current study, we screened the Ph.D. -12 phage display library against VEGFR-Fc fusion protein. And then an identified peptide with the sequence HRHTKQRHTALH was synthesized, and its anti-angiogenesis abilities were evaluated and confirmed *in vitro* and *in vivo*. This peptide holds great promise as an angiogenic inhibitor for the treatment of ophthalmic diseases caused by excessive angiogenesis.

## Materials and methods

### Phage display library and other reagents

A 12-mer phage display library (Ph.D. -12 phage display library) was purchased from New England Biolabs (Beverly, MA, USA), which was based on a combinatorial library of random dodecapeptides fused to a minor coat protein (pIII) of M13 phage. The VEGFR-Fc fusion protein was constructed by fusing the second Ig domain of VEGFR-1 with the third Ig domain of VEGF receptor-2 (VEGFR-2) to the constant region (Fc) of human IgG1,^27^ and expressed in Chinese hamster ovary cells in Shanghai General Hospital (Shanghai, China). *Escherichia coli* K12 ER2738 for amplification of phage and wild-type M13 phage clone were also purchased from New England Biolabs.

### Cell culture

Human umbilical vein endothelial cells (HUVECs) were obtained from BioWhittaker Inc. (Walkersville, MD, USA). The cells were cultured in the endothelial cell medium containing endothelial cell growth supplement (ScienCell, San Diego, CA, USA). The cells were used for the experiments at their second growth passage.

### Screening phage display library with VEGFR-Fc fusion protein

The procedure for positive screening the phage display library was mainly according to instructions of the manufacturer (New England Biolabs). Briefly, the plate was coated with 50 μg VEGFR-Fc fusion protein overnight at 4 °C with gentle agitation in a humidified container before blocking with phosphate buffer containing 1% bovine serum albumin for 1 h at 37 °C. A phage display library containing 1×10^12^ plague-forming unit (p.f.u.) phages was added to the coated plate and incubated at room temperature. After 1 h, the supernatant was removed. The plates were slapped face-down onto a clean paper towel and washed 16 times with TBST (0.1% Tween-20). The bound phages were eluted with a low pH buffer (2.2 M glycine, pH 2.2) and amplified in E. coli K12 ER2738 for two subsequent rounds of selection on VEGFR-Fc fusion protein. In the second and third affinity selections, the plates were washed with TBST (0.25% Tween-20) and TBST (0.5% Tween-20). After three positive panning rounds, the positively selected phages were amplified for the next negative selection. In the first round of negative panning, 1.5 μg of human IgG diluted in TBST (200 μl) was immobilized in protein A agarose resin. Subsequently, 1×10^10^ p.f.u. phages were mixed with the diluted IgG solution and incubated at room temperature for 20 min. Following incubation, the phages were transferred to the tube containing protein A agarose and further allowed to bind at room temperature for 15 min. Then, the tube was centrifuged at low speed and the supernatant was recovered. The above process was repeated for two additional rounds. The supernatant was titrated and the individual plaques were picked for DNA isolation and sequencing. Phages from selected clones were sequenced using an ABI machine. The above biopanning experiment was performed by Rx Biosciences, Ltd (Gaithersburg, MD, USA).

### Enzyme-linked immunosorbent assay for phage-binding activity

A 96-well plate was coated with 100 μl VEGFR-Fc fusion protein (10 μg ml^−1^) for each well at 4 °C overnight, then blocked with phosphate buffer containing 1% bovine serum albumin for 2 h at 37 °C. Phages were added to the wells and incubated at room temperature for 30 min. The amount of bound phages was detected with a horseradish peroxidase-conjugated anti-M13 antibody. After the addition of the substrate, the optical density (OD) of each well was measured at 405 nm using a Microplate Reader (Molecular devices, LLC, Sunnyvale, CA, USA). Wild-type M13 phage clone was served as a negative control. The enzyme-linked immunosorbent assay assay was conducted by Rx Biosciences, Ltd.

### Peptide synthesis and identification

Peptides were synthesized on a peptide synthesizer CS336X (CSBio Company Inc., Menlo Park, CA, USA) following the standard Fmoc strategy by Chengdu Nuoen Biotechnologies, Ltd (Chengdu, China). The fluorescein residue was introduced into the peptide by standard coupling of N-terminal-deprotected peptide with fluorescein-5-carboxylic acid. Peptide amides were cleaved from the resin with trifluoroacetic acid: triisopropylsilane: water (94: 4: 2, v/v/v) and were recovered by precipitation with ice-cold diethyl ether. Crude products were purified by high-performance liquid chromatography on a C18 column using a gradient of 20–80% acetonitrile/water (0.1% formic acid). The sequence of peptides was verified by electrospray mass spectrometry (Finnigan Iontrap Mass Analyzer, Thermo, Waltham, MA, USA).

### HUVEC proliferation assay

To evaluate the inhibition rate of endothelial proliferation stimulated by VEGF, HUVECs were placed into 96-well plates at 104 cells per well and incubated for 24 h at 37 °C in 5% CO_2_ (100 μl per well). The synthesized peptide diluted with endothelial cell growth supplement-endothelial cell medium (at varied concentrations from 0.95 to 3.85 μg μl^−1^) and equal volume of VEGF solution (40 ng ml^−1^) were mixed and incubated for 3 h at 37 °C. The peptide and VEGF mixture solution were added to each well of the plates (100 μl per well) and incubated for 96 h at 37 °C, and then they were treated with CCK-8 solution (25 μl per well). The absorbance was determined at 450 nm using a Microplate Reader (Molecular devices, LLC). The endothelial cell growth supplement-endothelial cell medium medium was used as a blank control. The VEGFR-Fc fusion protein was used as a positive control. The VEGF was used as a negative control. The cell growth rate was defined as OD sample/OD negative×100. This experiment was performed in triplicate by Chengdu Nuoen Biotechnologies, Ltd.

### *In vivo* study of angiogenesis using the chick chorioallantoic membrane assay

To determine anti-angiogenic activity *in vivo*, a chick chorioallantoic membrane (CAM) assay was performed by Shanghai General Hospital (Shanghai, China) as previously described.^28,29^ Two-day-old fertilized eggs (Shanghai Poultry Breeding Co. Ltd., Shanghai, China) were incubated at 37 °C and 60–70% relative humidity. After incubation for 5 days, a 1–2 cm^2^ window was opened and one sterilized silica gel O-type ring (0.5 cm inner diameter) was placed onto the CAM of every individual embryo, and phosphate buffer solution (PBS) or peptides (10, 25 or 50 μg μl^−1^) were added inside it. The eggs were incubated for another 48 h. The upper eggshell was removed and the area of capillary blood vessels was calculated. Capillaries were photographed under a stereomicroscope (Olympus, SZX16, Tokyo, Japan). The capillary area was calculated with Image-Pro Plus version 6.0.0.260 (Media Cybernetics, Inc., Rockville, MD, USA). The experiment was repeated three times.

### Animals

Sprague–Dawley male rats were purchased from Laboratorial Animal Center of Shanghai General Hospital. All animal experiments conformed to the Association for Research in Vision and Ophthalmology Statement for the Use of Animals in Ophthalmic and Vision Research. All of the experimental protocols were approved by the Animal Investigation Committee of Shanghai General Hospital, Shanghai Jiaotong University School of Medicine. All eyes of the rats were examined before experiments to exclude any pre-existing corneal diseases. One cornea of each rat was treated as an experimental eye. All the animals were anesthetized with an intraperitoneal injection of 30 mg kg^−1^ pentobarbital and received topical anesthetization with oxybuprocaine.

The experimental rats were randomly separated into three groups (eight for each group): (1) negative control group: PBS, (2) HRH peptide group: 10 μg μl^−1^, (3) nonspecific peptide group: 10 μg μl^−1^, treated with eye drops (10 μl per eye drop, four times per day). Corneal neovascularization (NV) was observed and photographed with a stereomicroscope (Olympus, SZX7) on days 3, 5, 7, 10 and 14 after operation. The experiments were performed in triplicate. All animal experiments were performed by Shanghai General Hospital (Shanghai, China).

### Corneal NV induced by alkali burn

One cornea of each Sprague–Dawley male rat in experimental groups was alkali burned. The rats were anesthetized as mentioned previously. A round filter paper of 4.0-mm diameter was soaked in 1 N NaOH for 20 s and applied on the central cornea for 40 s. After the removal of the filter paper from the cornea, the eye was rinsed with 10 ml of sterilized saline and ofloxacin ointment was instilled. Three different eye drops were topically applied to the injured corneas for 2 weeks, including PBS, peptide HRH and YIT.

The corneal NV was examined and photographed on the days 3, 5, 7, 10 and 14 after alkali burn. The area of corneal NV was calculated by the following formula: area (mm^2^)=*C*/12×3.1416×[*R*^2^−(*R*−*L*)^2^].^30,31^ The cornea image was divided into 12 clock hours. *C* is the clock hours of NV; *R* is the radius of the cornea; and *L* is the maximal vessel length. Both *R* and *L* of each cornea were measured five times *in vivo*, and the area of corneal NV was calculated accordingly.

### Rat corneal model of NV induced by intrastromal suture

Suture-induced cornea NV model was performed according to a previous report with some modifications.^32,33^ Briefly, 10-0 nylon was sutured into the stroma of the temporal half of the cornea in a radial direction. The stitch length was 1 mm, and outside stitching was 1 mm from the corneal limbus. Afterwards, treatment with eye drops continued for 2 week. The area of corneal NV was calculated by the following formula: area (mm^2^)=0.4×3.1416×[*R*^2^−(*R*−*L*)^2^]. *R* is the radius of the cornea and *L* is the maximal vessel length. Both *R* and *L* of each cornea were measured five times *in vivo*.

### Epitope mapping based on phage display

Peptides displayed on the identified phages were first scanned by the tools in the SAROTUP suite to exclude any possible target-unrelated peptides.^34–39^ The remaining peptides were then mapped back to the surface of VEGF and placenta growth factor (PlGF) based on its crystal structure (PDB: 1FLT, 2XAC, 3V2A, 2X1W and 1RV6) using the EpiSearch program by default parameters.^40^ The mapping result with the highest score was united to make the epitope on VEGF and PlGF recognized by VEGFRs.

### Data analysis and statistics

Statistical analyses were performed by SPSS for Windows version 19.0 (SPSS Inc., Chicago, IL, USA). The data were analyzed using Student’s *t*-test and one-way analysis of variance. A *P*-value <0.05 was considered statistically significant.

## Results

### Affinity selection of phages binding with VEGFR-Fc fusion protein and sequence analysis

**Figure 1:**
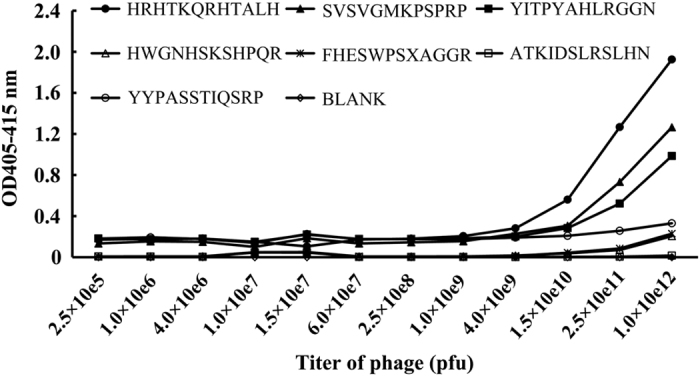
Binding ability of phage clones to VEGFR-Fc fusion protein. The bound phages were detected by phage enzyme-linked immunosorbent assay. The VEGFR-Fc fusion protein was incubated with phages at various titers ranging from 2.5×10^5^ to 1×10^12^ p.f.u. at room temperature for 30 min. The OD value at 405–415 nm was measured. Wild-type M13 phage was used as a negative control (data not shown).

We screened Ph.D. -12 phage display library of peptides binding with VEGFR-Fc fusion protein. After the last round of panning, 95 clones were randomly picked out and the peptide sequences were identified. Among the identified sequences, there were 11 peptides appearing at least twice and therefore they were considered as consensus sequences (Table 1). In addition, the binding activity of selected phage clones was estimated by enzyme-linked immunosorbent assay. Wild-type M13 phage with no inserted peptide was used as a negative control. The results revealed that selected clones displaying HRHTKQRHTALH (peptide HRH), YITPYAHLRGGN (peptide YIT) and SVSVGMKPSPRP (peptide SVS) had greater reactivity for VEGFR-Fc fusion protein compared with other phage clones (Figure 1). These three sequences were selected for further bioinformatic analysis. To exclude any possible target-unrelated peptides (TUPs) from biopanning results, all peptides were scanned using SAROTUP.^34–37^ The TUPScan tool in SAROTUP suggested that the SVS peptide, appearing in the output of many other phage display experiments,^41^ was a binder to immunoglobulin Fc region or unrelated antibodies.^42^ In addition, this peptide was reported to be very likely a propagation-related TUP.^43^ Accordingly, the peptide was excluded from further studies. The MimoSearch tool in the SAROTUP suite indicated that the YIT peptide was selected by three completely different targets. Thus, this peptide is unlikely to be a specific binder. The left HRH peptide was then mapped back to the surface of VEGF and PlGF using the EpiSearch program by default parameters.^40^ The solutions with the highest scores were chosen, and their results were united to make the epitope on PlGF and VEGF recognized by VEGFR-1 and VEGFR-2. As shown in Table 2, we found that three to six residues in the epitope mapping results also appeared in the interfaces of VEGF-A/VEGFR-1, VEGF-B/VEGFR-1 and VEGF-C/VEGFR-2 complexes, respectively (see the residues in bold), whereas no residues overlapped with those of PlGF/VEGFR-1 and VEGF-A/VEGFR-2 complexes. In the following experiments, the HRH peptide was synthesized, whereas the YIT peptide was also synthesized and used as a peptide control.

### Peptide HRH inhibits the proliferation of HUVEC

**Figure 2:**
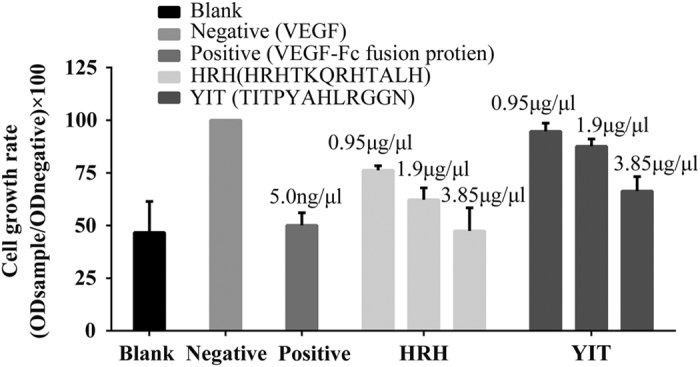
Inhibition rate of peptide HRH at various concentrations. The endothelial cell growth supplement-endothelial cell medium medium was used as a blank control. The VEGF was used a negative control. The VEGFR-Fc fusion protein was used as a positive control. The YIT peptide was used as a nonspecific peptide control. The absorbance was determined at 450 nm. The cell growth rate was defined as OD sample/OD negative ×100.

The HUVEC exerts major influences on the sprout and growth of blood vessel and is commonly used to assess anti-angiogenesis activity *in vitro*. The inhibitory ability of peptide HRH on endothelial proliferation stimulated by VEGF was investigated by HUVEC. Results of the proliferation assay revealed that HUVECs treated with the HRH peptide and VEGFR-Fc fusion protein have a lower cell growth rate than those treated with only VEGF (Figure 2), which demonstrated that peptide HRH efficiently suppressed proliferation of HUVEC under VEGF stimulation. As shown in Figure 2, peptide HRH affected endothelial cell proliferation in a dose-dependent manner, and HUVECs handled with peptide HRH grew slower than those treated with the control peptide YIT at each concentration.

### Peptide HRH suppresses angiogenesis in the CAM

**Figure 3:**
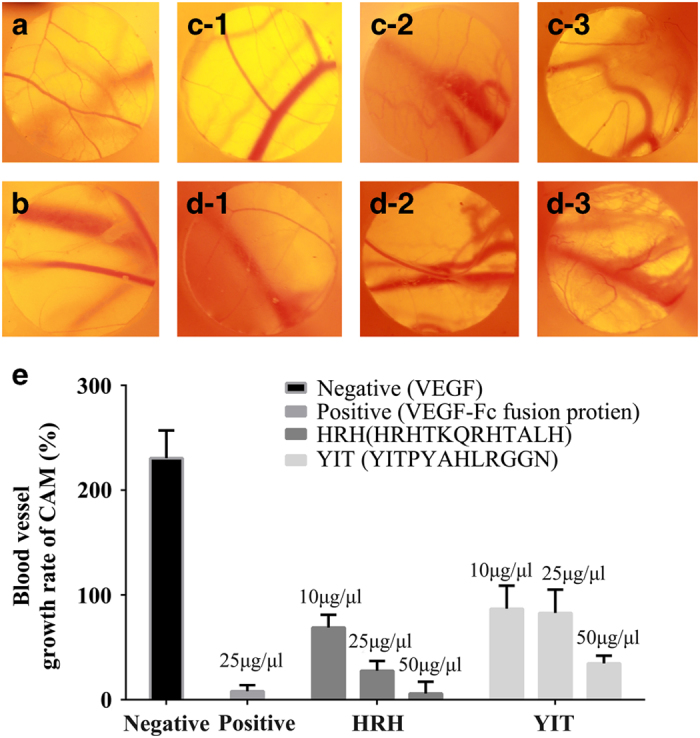
The effect of peptide HRH on inhibition of angiogenesis in chorioallantoic membrane. Capillaries were photographed: (**a**) PBS; (**b**) VEGFR-Fc fusion protein; (**c**-1) 10 μg μl^−1^ YIT; (**c**-2) 25 μg μl^−1^ YIT; (**c**-3) 50 μg μl^−1^ YIT; (**d**-1) 10 μg μl^−1^ HRH; (**d**-2) 25 μg μl^−1^ HRH; (**d**-3) 50 μg μl^−1^ HRH. (Magnification ×10). (**e**) Blood vessel growth rate of CAM.

To examine the *in vivo* anti-angiogenic activity, the CAM assay was carried out to determine the influence of peptide HRH on angiogenesis. Blood vessel growth rate is reflected by the increased vessel area after 48 h compared with original area. As shown in Figure 3, CAMs treated with the HRH peptide at the concentration of 10–50 μg μl^−1^ demonstrate obvious avascular area and the decrease in the area of capillaries in the treated area (Figures 3d-1–d-3) in contrast to CAMs treated with PBS (Figure 3a). The area of newly-formed blood vessels was significantly decreased in a dose-dependent manner over the range from 10 to 50 μg μl^−1^ without inflammation (Figures 3d-1–d3 and e). With the increase of the concentration, the difference between the inhibition of angiogenesis of peptide HRH and peptide YIT is much more significant (Figures 3c-1–c-3). When the concentration of the HRH peptide reached 50 μg μl^−1^, its inhibition capabilities on blood vessel growth can rival those of the VEGFR-Fc fusion protein. These data suggest that the HRH peptide can efficiently suppress angiogenesis in chicken embryos.

### Peptide HRH inhibits corneal NV in alkali-burnt rat corneal model

**Figure 4:**
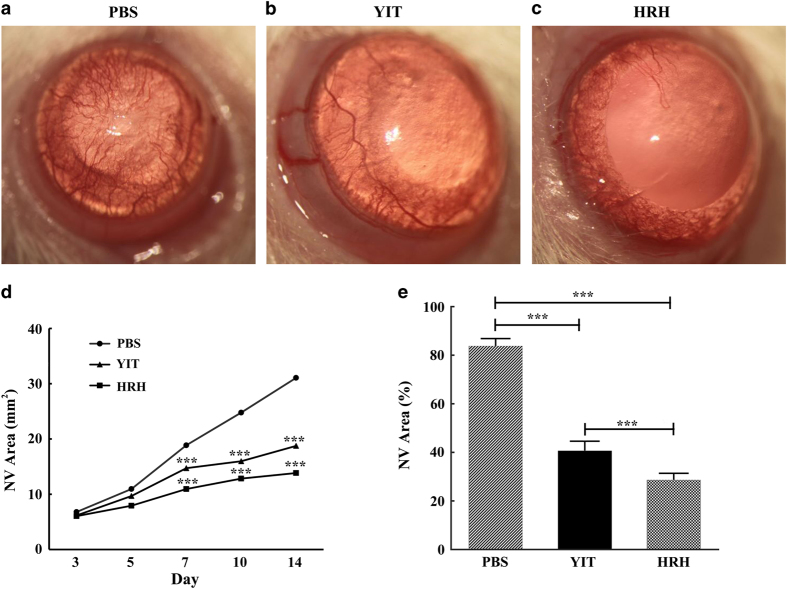
Effects of peptide HRH on corneal NV induced by alkali burn. The corneal NV was measured and photographed with a stereomicroscope after alkali burn. (**a**–**c**) Images of burned eyes treated with PBS, peptide YIT and peptide HRH at the concentration of 10 μg μl^−1^, respectively. (**d**, **e**) The total NV area was measured and analyzed. (data are presented as mean±s.e.m., *n*=8 in each group, ***P<0.001).

The results of CAM assays encouraged us to determine whether peptide HRH may also affect corneal NV. Alkali-burned cornea induced obvious neovasculature, and the blood vessels in the PBS group almost covered the whole corneal stroma (Figure 4a). Most part of corneal stroma did not have blood vessels in the HRH group (Figure 4c) compared to the PBS group (Figure 4a) and the YIT group (Figure 4b). From days 3 to 14, the growth of the vessel in HRH group slowed down and the NV area increased slowly (Figure 4d). Compared to the PBS group and the YIT group, the area of corneal NV, which was treated with peptide HRH, was significantly reduced (***P<0.001, Figure 4e).

### Peptide HRH depresses corneal NV in suture-induced rat corneal model

**Figure 5:**
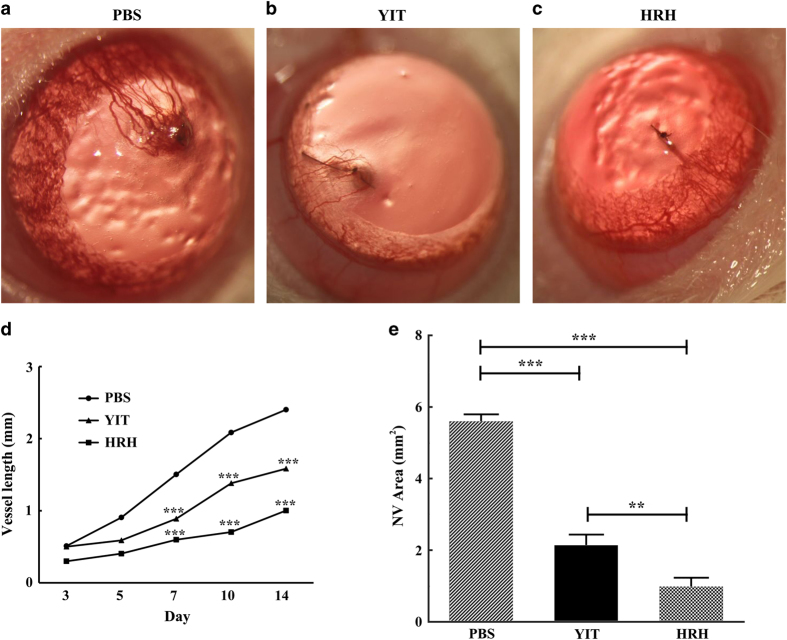
Effects of peptide HRH on NV induced by intrastromal suture. Corneal NV was measured and photographed with a stereomicroscope after intrastromal suture. (**a**–**c**) Images of group PBS, group peptide YIT, peptide HRH at the concentration of 10 μg μl^−1^. (**d**, **e**) The length of the longest vessel and the total corneal NV area were measured and analyzed. (data are presented as mean±s.e.m., *n*=8 in each group, ***P<0.001, **0.001<*P*<0.003).

Further investigation was performed to test the effect of peptide HRH on suture-induced corneal NV through observing its inhibitory effect on the growth of blood vessels. Compared with those treated with PBS and peptide YIT, corneal NV was significantly inhibited by topical administration of peptide HRH (*P*<0.001; Figure 5). In comparison to controls, the vessel invasion area was significantly reduced.

## Discussion

In this study, we utilized a subtractive biopanning strategy to isolate VEGFR-binding peptides from a phage-displayed 12-mer peptide library. One peptide with the sequence HRHTKQRHTALH was identified and characterized. The HUVEC proliferation experiment demonstrated that peptide HRH was able to block the generation of new blood vessels activated by VEGF *in vitro*. And the peptide could markedly reduce *in vivo* angiogenic activity dose-dependently in the CAM assay. In addition, the HRH peptide was also proved to inhibit corneal NV in an alkali-burnt rat corneal model and a suture-induced rat corneal model.

### Potential anti-angiogenesis mechanism of peptide HRH

The VEGF is a critical proangiogenic factor in NV, which acts through its receptors, VEGFR-1 and VEGFR-2.^5^ The VEGFR-Fc fusion proteins such as aflibercept^27^ and conbercept^3^ consist of the second Ig domain of VEGFR-1 and the third Ig domain of VEGFR-2. These fusion proteins have a broader affinity for VEGF molecules and have shown significant therapeutic effects on the treatment of AMD.^3,44^ In this study, we used VEGFR-Fc fusion protein as the target molecule and aimed to identify peptide ligands that can bind to VEGFR-1 and VEGFR-2. These peptides may have more advantages and better development prospects than those selected by natural VEGFR-1 or VEGFR-2. We found that the HRH peptide had the highest affinity to the VEGFR-Fc fusion protein. The epitope analysis results of the peptide were compared with genuine VEGFR-binding sites on ligands. As many as six residues in the epitope mapping result also appeared in the site that VEGF-B is bound to VEGFR-1. The significant overlapping suggests that the HRH peptide is a mimotope that mimicks the binding sites of VEGFR-1 on the VEGF-B. We found that three residues in the epitope mapping results also appeared in the interfaces of VEGF-A/VEGFR-1 and VEGF-C/VEGFR-2 complexes, respectively (see the residues in bold in Table 2), whereas no residues overlapped with those of PlGF/VEGFR-1 and VEGF-A/VEGFR-2 complexes. On the basis of the above results, we infer that the HRH peptide can competitively inhibit the binding of VEGF-A and VEGF-B to VEGFR-1, and VEGF-C to VEGFR-2, leading to its anti-angiogenesis activity. The exact biochemical mechanism of the HRH peptide and the effect of the peptide on VEGFRs intracellular pathways remain to be further studied.

### Promising application prospects of anti-angiogenesis peptide HRH

Anti-angiogenic agents are effective in the treatment of human cancers and fundus neovascular diseases.^45^ In combination with chemotherapy, they prolong the life of patients with certain types of cancers.^46^ Furthermore, anti-angiogenic treatments for ocular diseases characterized by growth of new blood vessel are commonly used,^45^ and have greatly reduced the incidence of blindness from exudative AMD.^3^ Since 2004, five angiogenesis inhibitors targeting VEGF have been introduced to ophthalmology, namely, pegaptanib, ranibizumab, bevacizumab, aflibercept and conbercept.^3^ These drugs produce promising therapeutic effects in patients with neovascular AMD. It has also been reported that anti-angiogenic therapy provides a possible therapeutic choice for the treatment of diet-induced obesity and metabolic complications.^47–51^ Given this, it is convinced that this novel angiogenic inhibitor will have wide application prospects.

## Conclusion

In the present study, we successfully discovered a novel anti-angiogenesis peptide from a random phage peptide library, which can significantly restrain the development of new blood vessels *in vitro* and *in vivo*, and inhibit corneal NV in an alkali-burnt rat corneal model and a suture-induced rat corneal model. The peptide provides a new candidate for suppressing the occurrence of new blood vessels. Peptide HRH holds great promise as a therapeutic lead agent for the treatment of complex angiogenesis-related pathological disorders.

## Figures and Tables

**Table 1:** Phage-displayed peptide sequences selected by VEGFR-Fc fusion protein

*Peptide*	*Sequence*	*Phage clone*	*Occurrence*
HRH	HRHTKQRHTALH	B12, C12, E5, E12, G8, H12	6/33
YIT	YITPYAHLRGGN	B7, C9, D7, D12	4/33
SVS	SVSVGMKPSPRP	B1, B11, D5, G12, H3	5/33
FHE	FHESWPSXAGGR	B4, C6, F9	3/33
TMG	TMGFTAPRFPHY	E1, H4, H5	3/33
TSD	TSDIKSRSPHHR	A5, D6	2/33
QTG	QTGHWNAEWHTR	A6, H7	2/33
ATK	ATKIDSLRSLHN	A10, F2	2/33
YYP	YYPASSTIQSRP	C4, F1	2/33
HWG	HWGNHSKSHPQR	D2, E11	2/33
SHP	SHPWNAQRELSV	E2, G4	2/33

**Table 2:** Comparing epitope mapping result of HRH peptide with genuine VEGFR-binding site on ligand

*Receptor*	*Ligand*	*Complex*	*HRH peptide mapping results*	*PDB sum interface*
VEGFR-1	VEGF-A	1FLT	K16, **Q22**, R23, H27, T31, L32, Q37, **K48**, R56, T71, T77, Q79, R82, **Q89**, H90, L97, Q98, H99, K101	F17, M18, Y21, **Q22**, Y25, **K48**, D63, G65, L66, M81, I83, H86, **Q89**, I91, E103, C104, R105, P106
VEGFR-1	VEGF-B	2XAC	**T22**, **T25**, L35, T36, L39, T42, A44, K45, Q46, L47, **Q79**, **L81**, R84, **Q89**, **L90**	S16, W17, Y21, **T22**, **T25**, Q27, V48, P62, D63, L66, **Q79**, **L81**, S88, **Q89**, **L90**, C103, P105
VEGFR-1	PlGF	1RV6	R35, A36, L37, R39, L63, R64, T66, T81, A82, Q106, H107, R109	F25, Q26, W29, G30, Y33, M54, S56, G70, D71, L74, N73, Q87, L89, I91, P97, Y99, C112, R113, P114
VEGFR-2	VEGF-A	3V2A	H27, T31, R56, L66, T71, L97, Q98, H99, K101, R105	P40, D41, I43, E44, K48, M81, I83, P85, H86, Q89
VEGFR-2	VEGF-C	2X1W	**R127**, K128, **Q130**, R134, A137, R161, L171, Q172, T176, H206, **R210**	T116, L119, D123, W126, **R127**, **Q130**, K142, T148, N149, F151, K153, N167, S168, E169, G170, F186, I188, V190, P191, L192, G195, P196, **R210**, K214, L215

Abbreviations: PlGF, placenta growth factor; VEGF, vascular endothelial growth factor; VEGFR, VEGF receptor.

Overlapping residues were in bold.
